# Supplementary material for: One-third of amenorrheic transmasculine people on testosterone ovulate
Source: Cell Rep Med. 2024 Feb 22;5(3):101440. doi: 10.1016/j.xcrm.2024.101440 (PMC10982961; doi:10.1016/j.xcrm.2024.101440)
Supplement: Document S1. Table S1 [file mmc1.pdf]

**Cell Reports Medicine, Volume *5***

## Supplemental information

# One-third of amenorrheic transmasculine people on testosterone ovulate

**Joyce D. Asseler, Julieta S. del Valle, Susana M. Chuva de Sousa Lopes, Marieke O. Verhoeven, Mariette Goddijn, Judith A.F. Huirne, and Norah M. van Mello**

Table S1. Data on prior GnRHa users of study cohort, related to Table 1.

| | Age at GAS, years | Age at start GnRHa, years | Total time on GnRHa, months | Indication GnRHa | Tanner stage at time start GnRHa | Time of GnRHa cessation prior to GAS, months | Histological signs of recent ovulatory activity |
|---|---|---|---|---|---|---|---|
| 1 | 20 | 14 | 40 | PS | Unknown | 30 | Yes, corpus luteum and corpus albicans |
| 2 | 19 | 14 | 19 | PS | M4P5 | 35 | Yes, corpus luteum and corpus albicans |
| 3 | 20 | 16 | 14 | PS | M5P5 | 22 | Yes, corpus albicans |
| 4 | 21 | 17 | 9 | PS | M5P5 | 40 | Yes, corpus luteum |
| 5 | 20 | 17 | 11 | PS | M5P5 | 23 | Yes, corpus luteum |
| 6 | 18 | 13 | 47 | PS | M3P3 | 9 | No |
| 7 | 18 | 16 | 19 | PS | M5P5 | 8 | No |
| 8 | 21 | 17 | 26 | PS | M5P5 | 18 | No |
| 9 | 19 | 17 | 12 | PS | M5P5 | 25 | No |
| 10 | 28 | 26 | 4 | B | N/a | 18 | No |
| 11 | 19 | 11 | 49 | PS | M4P5 | 42 | No |
| 12 | 21 | 17 | 12 | PS | M5P5 | 40 | No |
| 13 | 19 | 16 | 36 | PS | Unknown | 7 | No |
| 14 | 19 | 12 | 50 | PS | M3P2 | 31 | No |
| 15 | 18 | 12 | 55 | PS | M3P4 | 19 | No |

Abbreviations used in table: GnRHa = gonadotropin-releasing hormone agonist, PS = puberty suppressant, N/a = not applicable, B: persistent blood loss.
