# Supplementary material for: One-third of amenorrheic transmasculine people on testosterone ovulate
Source: Cell Rep Med. 2024 Feb 22;5(3):101440. doi: 10.1016/j.xcrm.2024.101440 (PMC10982961; doi:10.1016/j.xcrm.2024.101440)
Supplement: Document S2. Article plus supplemental information [file mmc2.pdf]

Cell Reports
Medicine

Report

One-third of amenorrheic transmasculine people on testosterone ovulate

Graphical abstract

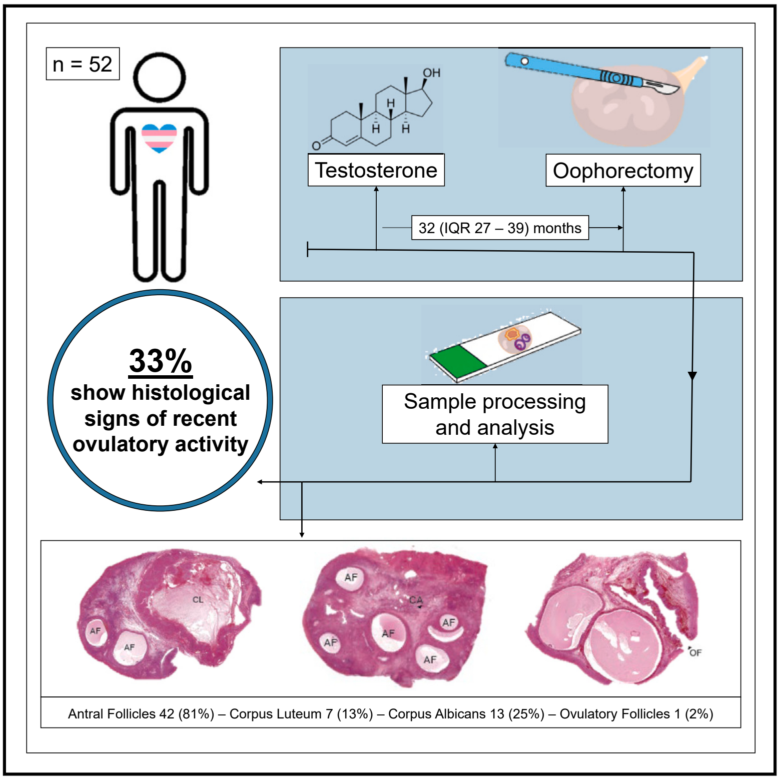

Authors

Joyce D. Asseler, Julieta S. del Valle, Susana M. Chuva de Sousa Lopes, Marieke O. Verhoeven, Mariette Goddijn, Judith A.F. Huirne, Norah M. van Mello

Correspondence

j.d.asseler@amsterdamumc.nl (J.D.A.), n.vanmello@amsterdamumc.nl (N.M.v.M.)

In brief

Asseler et al. report a surprising finding in transmasculine and gender-diverse individuals undergoing testosterone therapy. Contrary to common belief, their study reveals ovulatory activity in 33% of amenorrheic participants during gender-affirming oophorectomy. This challenges assumptions about anovulation during amenorrhea, highlighting the need for contraception in this demographic.

Highlights

- 33% of amenorrheic TGD people on testosterone show recent ovulatory activity
- These findings challenge the assumption that amenorrhea equals anovulation
- Contraception remains vital for preventing pregnancy in this demographic

Asseler et al., 2024, Cell Reports Medicine *5*, 101440
March 19, 2024 © 2024 The Authors.
https://doi.org/10.1016/j.xcrm.2024.101440

CellPress

Cell Reports Medicine

CellPress
OPEN ACCESS

Report

One-third of amenorrheic transmasculine people on testosterone ovulate

Joyce D. Asseler,[1,2,3,*] Julieta S. del Valle,[4] Susana M. Chuva de Sousa Lopes,[4,5] Marieke O. Verhoeven,[1,3] Mariette Goddijn,[3,6] Judith A.F. Huirne,[1,3] and Norah M. van Mello[1,2,3,7,*]
[1]Amsterdam UMC, Location Vrije Universiteit Amsterdam, Department of Obstetrics and Gynaecology, Amsterdam, the Netherlands
[2]Amsterdam UMC, Centre of Expertise on Gender Dysphoria, Amsterdam, the Netherlands
[3]Amsterdam Reproduction and Development Research Institute, Amsterdam, the Netherlands
[4]Leiden University Medical Center, Department of Anatomy and Embryology, Leiden, the Netherlands
[5]Ghent University Hospital, Department of Reproductive Medicine: Ghent-Fertility and Stem Cell Team (G-FAST), Ghent, Belgium
[6]Amsterdam UMC Location University of Amsterdam, Center for Reproductive Medicine, Department of Obstetrics and Gynaecology, Amsterdam, the Netherlands
[7]Lead contact
*Correspondence: j.d.asseler@amsterdamumc.nl (J.D.A.), n.vanmello@amsterdamumc.nl (N.M.v.M.)
https://doi.org/10.1016/j.xcrm.2024.101440

SUMMARY

Transmasculine people usually reach amenorrhea within 6 months of adequate testosterone treatment. It is often assumed that no ovulation occurs during amenorrhea. However, in this study, we report recent ovulatory activity in amenorrheic transmasculine people on testosterone therapy at gender-affirming oophorectomy. Histological signs of recent ovulatory activity, including the presence of ovulatory follicles, corpus luteum, and corpus albicans, are observed in 17 of 52 individuals (33%). This is not significantly correlated to the duration, testosterone serum levels, or type of testosterone used. These results suggest that amenorrhea does not equal anovulation in transmasculine people on adequate testosterone therapy, emphasizing the importance of contraception for people who engage in sexual activity that can result in pregnancy.

INTRODUCTION

Transgender and gender-diverse (TGD) people experience a persistent incompatibility between their gender identity and their birth-assigned sex.[1] In this study, we used the term transmasculine people to describe TGD people assigned female at birth but who identify otherwise.

Treatment options for transmasculine people are gender-affirming hormone treatment with testosterone and an array of gender-affirming surgeries (GASs) such as a hysterectomy and oophorectomy. Testosterone will lead to desirable masculinization of the body, such as deepening of the voice, facial hair growth, and a redistribution of muscle and fatty tissue. However, the most anticipated effect of testosterone is to achieve amenorrhea,[2] which is often achieved within 3–6 months.[3,4]

The general assumption is that testosterone induces hypothalamic-pituitary-gonadal suppression, resulting in anovulation and amenorrhea.[5] However, impact on the ovarian cycle remains unclear. Since escape ovulations resulting in unplanned pregnancies have been described in transmasculine people on testosterone,[6] contraceptive counseling is already included in recommended guidelines, such as the Standards of Care v.8 (SOC 8).[1] Furthermore, there are also data reported on intendedly pregnant transmasculine people who conceived while still amenorrheic from prior testosterone use.[7]

Even though the above-mentioned ovulatory events are hypothesized to be induced by inadequate testosterone serum levels, a recent study showed significant as well as transient rises in pregnanediol-3-glucuronide in urine samples of transmasculine people on continuous adequate testosterone treatment.[5] Pregnanediol-3-glucuronide, a metabolite of progesterone, reaches its maximum level after ovulation and is often used for non-invasive confirmation of ovulation in cycle charting.[5] Moreover, the presence of dominant follicles has been noted during trans-abdominal ultrasound screening, and live ovulations—a mature follicle rupturing—have been observed during laparoscopic GAS in amenorrheic transmasculine people within the serum testosterone reference range at our center, suggesting the ovary may still cycle.

This study aimed to determine the presence of recent ovulatory activity in ovaries obtained at gender-affirming oophorectomy in amenorrheic transmasculine people on adequate testosterone therapy.

RESULTS

Between May 2019 and May 2022, 52 participants were included in this study cohort. For a flowchart of patient inclusion, see Figure 1. Baseline characteristics are presented in Table 1. The median age of participants was 22 years old (interquartile range

This is an open access article under the CC BY license (http://creativecommons.org/licenses/by/4.0/).

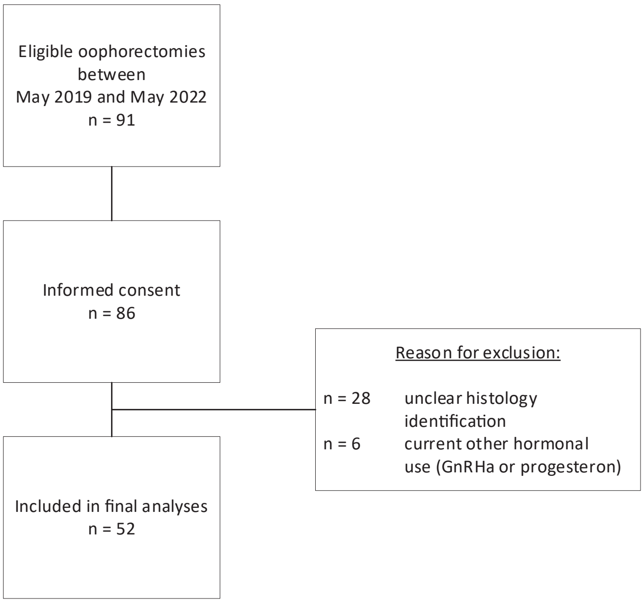

Figure 1. Flowchart of participant inclusion

[IQR] 20–28), and they were largely within a healthy BMI range with a median of 23.9 (IQR 21.6–27.2). A quarter of the cohort were smokers. Pre-operative American Society of Anesthesiologists (ASA) scores were reported for 30 participants, and two of them had an ASA score of III (based on severe systemic disease) unrelated to gender dysphoria or indication for surgery. Median time on testosterone treatment was 32 months (IQR 27–39). The majority of the cohort was using intramuscular injections at time of GAS, of which 40% used Nebido, 42% used Sustanon, and 17% were using AndroGel. Testosterone levels were taken randomly throughout the 12 month period preceding GAS and were never below physiological values. Hormone serum levels within 12 months prior to GAS were available for 43 transmasculine people, resulting in a median testosterone serum level of 21 nmol/L (IQR 13–29).

Small antral follicles were observed in the majority of the ovaries (81%). Furthermore, a total of 21 signs of recent ovulatory activity were observed in the ovaries, including one ovulatory follicle (2%), 7 corpus luteum (13%), and 13 corpus albicans (25%) in 17 transmasculine individuals (33%) (see Figure 2 and Table 2). The occurrence of these histological features was not statistically significantly correlated with the duration of testosterone treatment (Mann-Whitney U: $p = 0.310$), testosterone serum level (Fisher's exact test: $p = 0.246$), or the type of testosterone treatment (chi-squared test: see Table 2).

Gonadotropin-releasing hormone agonist (GnRHa) was previously used by 15 transmasculine people with a mean of 23 months (SD 12) of cessation prior to GAS. Most often, the indication for GnRHa use was puberty suppression. The majority of prior GnRHa users (9/15) were $\geq$Tanner stage 4 at the time of GnRHa initiation. In this group, five people showed histological signs of recent ovulatory activity. For further details on the prior GnRHa users, see Table S1.

Table 1. Baseline characteristics of study cohort

	N = 52
Age, median (IQR), years	22 (20–28)
BMI, median (IQR), kg/m^2	23.9 (21.6–27.2)
Smoker, n (%)	13 (25)
ASA classification, n (%)[a]	
I	9 (17)
II	19 (37)
III	2 (4)
Testosterone use, median (IQR), months	32 (27–39)
Type of testosterone used currently, n (%)	
AndroGel	9 (17)
Sustanon	22 (42)
Nebido	21 (40)
Prior GnRHa use, n (%)[b]	15 (29)
Time off GnRHa, mean (SD), months	24 (12)
Most recent laboratory hormone status prior to GAS,[c] median (IQR)	
Estradiol, pmol/L	119 (76–133)
LH, U/L	1.10 (0.10–4.00)
Testosterone, nmol/L	21 (13–29)

Data are expressed in mean (SD), median (IQR), and numbers (%). ASA, American Society of Anesthesiologists; BMI, body mass index; GnRHa, gonadotropin-releasing hormone agonist; LH, luteinizing hormone; GAS, gender-affirming surgery; SD, standard deviation; IQR, interquartile range.
[a]Data presented for n = 30
[b]For more information on prior GnRHa use, see Table S1.
[c]Data presented for n = 43, maximum 12 months old.

DISCUSSION

In this study, we observed that 33% of amenorrheic transmasculine participants on adequate testosterone treatment had histological signs of recent ovulatory activity in the form of corpus luteum, corpus albicans, and one ovulatory follicle. The occurrence of recent ovulatory activity was not statistically significantly correlated to the duration, serum level, or type of testosterone treatment.

Previously, seven studies reported on histological signs of ovulation in a total of 139 transmasculine people, varying from 6% to 84%,[8–14] and one older study even reported 100% presence of corpus albicans.[13] In these studies, it was unclear whether participants were on testosterone treatment and for how long. Moreover, it was not reported if testosterone treatment was interrupted prior to GAS. This may explain why these studies, especially the studies performed >10 years ago, reported even higher ovulatory signs compared to this study.

There are several other studies following GAS that reported on follicular count in histological samples without providing specific data on ovulatory stigmata, as their aim was to detect benign and/or malignant pathology and not histological signs of ovulation.[15–20] Therefore, the lack of ovulatory stigmata reported does not imply that there were none.

The evidence for recent ovulatory activity in transmasculine people at GAS implies that there is a risk for unplanned

CellPress
OPEN ACCESS

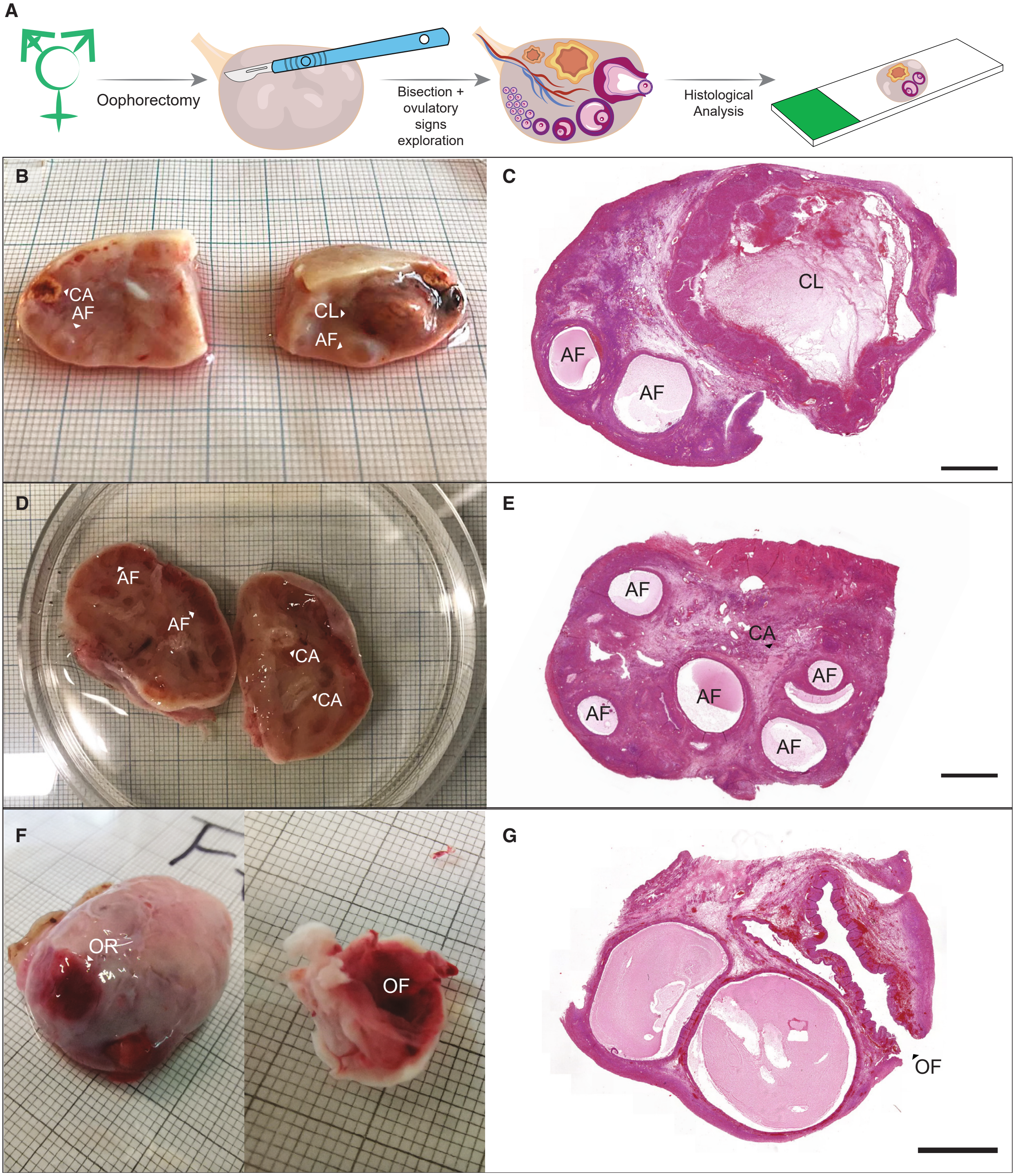

Figure 2. Ovaries from transmasculine participants (n = 3) and associated H&E-stained histological sections

(A) Transmasculine ovarian analysis.

(B) Bisected ovary with presence of corpus luteum (CL), corpus albicans (CA), and several antral follicles (AFs).

(C) H&E-stained ovarian section containing CL and two AFs.

(legend continued on next page)

Table 2. Histological features of follicular and recent ovulatory activity and its correlation to type of testosterone treatment

	Subjects included in analysis, n = 52 (%)	AndroGel, n = 9	Sustanon, n = 22	Nebido, n = 21	p value[a]
Small antral follicles	42 (81)	7	16	19	0.596
Ovulatory follicles	1 (2)[b]	0	1	0	0.499
Corpus luteum	7 (13)[b]	3	2	2	0.158
Corpus albicans	13 (25)[b]	2	5	6	0.887

Data are expressed in numbers (%).
[a]Chi-squared test.
[b]A total of 21 occurrences of recent ovulatory activity were observed in 17 individuals (33%), as participants could display multiple histological signs of recent ovulation.

pregnancy in amenorrheic transmasculine people on adequate testosterone. Unplanned pregnancy and the subsequent decisions have grave physical and mental impacts on the individual, their loved ones, and even their caregivers. Since TGD people have limited access to reproductive healthcare in the world,[21] it is of particular importance that contraceptive counseling remains an essential part of transgender healthcare, especially since testosterone is considered teratogenic.

Limitations of the study

A strength of this study is the unique study cohort, providing insights on the role of testosterone in ovarian activity. Our results challenge the current understanding of the effects of testosterone on the menstrual cycle (complete suppression of the gonadal-hypothalamic axes) and show that amenorrhea in fact does not equal anovulation in transmasculine people on testosterone.

One limitation of the study is the time gap between obtaining hormonal serum laboratory results and GAS. We included participants whose hormone levels were measured within 12 months prior to GAS, assuming that their testosterone levels remained stable due to their prolonged use, as is clinical practice. This approach aligns with the recommendations of SOC 8,[1] which suggest no more than (bi-)annual laboratory monitoring once a steady adult maintenance dose of testosterone is achieved after 1 year of use. Furthermore, all participants received their ongoing prescriptions from our clinic, indicating a continuous use of medication. However, other serum hormones may fluctuate more than testosterone, possibly being less reliable if analyzed long before surgery. For future research, we would suggest that hormonal levels of testosterone, luteinizing hormone, estradiol, and progesterone be determined on the day of GAS for a more accurate representation of the hormonal status in a person.

Conclusion

In this cohort, 33% of participants showed recent histological signs of ovulation unrelated to duration, serum level, or type of testosterone. Amenorrhea does not equal anovulation in transmasculine people on adequate testosterone therapy, emphasizing the importance of contraception for people who engage in sexual activity that can result in pregnancy.

STAR★METHODS

Detailed methods are provided in the online version of this paper and include the following:

- KEY RESOURCES TABLE
- RESOURCE AVAILABILITY
  - Lead contact
  - Materials availability
  - Data and code availability
- EXPERIMENTAL MODEL AND STUDY PARTICIPANT DETAILS
  - Study design and population
  - Ethical approval
- METHOD DETAILS
  - Laboratory procedures
- QUANTIFICATION AND STATISTICAL ANALYSIS
  - Imaging and quantification criteria
  - Statistical analysis

SUPPLEMENTAL INFORMATION

Supplemental information can be found online at https://doi.org/10.1016/j.xcrm.2024.101440.

ACKNOWLEDGMENTS

J.S.d.V. and S.M.C.d.S.L. declare funding by the European Research Council (ERC-CoG-2016-725722 OVOGROWTH) and a Novo Nordisk Foundation grant (reNEW NNF21CC0073729).

AUTHOR CONTRIBUTIONS

Conceptualization, J.D.A. and N.M.v.M.; methodology, J.D.A., J.S.d.V., S.M.C.d.S.L., and N.M.v.M.; investigation, J.D.A. and J.S.d.V.; formal analysis, J.D.A., J.S.d.V., S.M.C.d.S.L., and N.M.v.M.; data curation, J.D.A. and J.S.d.V.; writing – original draft, J.D.A. and J.S.d.V.; writing – reviewing & editing, S.M.C.d.S.L., M.O.V., M.G., J.A.F.H., and N.M.v.M.; supervision, S.M.C.d.S.L. and N.M.v.M.

(D) Bisected ovary showing presence of two CAs and several AFs.
(E) H&E-stained ovarian section containing part of a CA and several AFs.
(F) From left to right: ovarian surface showing the ovarian rupture (OR) site where the ovulation occurred.
(G) H&E-stained ovarian section demonstrating an ovulatory follicle (OF). Scale bar: 2,000 μm.

CellPress
OPEN ACCESS

DECLARATION OF INTERESTS

M.G. and M.O.V. declare that their Department of Reproductive Medicine received research and educational grants from Guerbet, Merck, and Ferring (all location VUmc), outside the scope of the submitted work. J.A.F.H. received grants from ZonMw, Samsung, and PlantTec MedicalGmbH outside the scope of the submitted work.

Received: July 20, 2023
Revised: September 27, 2023
Accepted: January 31, 2024
Published: February 22, 2024

REFERENCES

1. Coleman, E., Radix, A.E., Bouman, W.P., Brown, G.R., de Vries, A.L.C., Deutsch, M.B., Ettner, R., Fraser, L., Goodman, M., Green, J., et al. (2022). Standards of Care for the Health of Transgender and Gender Diverse People, Version 8. Int. J. Transgend. Health *23*, S1–s259. https://doi.org/10.1080/26895269.2022.2100644.
2. Masumori, N., Baba, T., Abe, T., and Niwa, K. (2021). What is the most anticipated change induced by treatment using gender-affirming hormones in individuals with gender incongruence? Int. J. Urol. *28*, 526–529. https://doi.org/10.1111/iju.14499.
3. Ahmad, S., and Leinung, M. (2017). The Response of the Menstrual Cycle to Initiation of Hormonal Therapy in Transgender Men. Transgend. Health *2*, 176–179. https://doi.org/10.1089/trgh.2017.0023.
4. Defreyne, J., Vanwonterghem, Y., Collet, S., Iwamoto, S.J., Wiepjes, C.M., Fisher, A.D., Schreiner, T., Heijer, M.D., and T'Sjoen, G. (2020). Vaginal bleeding and spotting in transgender men after initiation of testosterone therapy: A prospective cohort study (ENIGI). Int. J. Transgend. Health *21*, 163–175. https://doi.org/10.1080/26895269.2020.1719951.
5. Taub, R.L., Ellis, S.A., Neal-Perry, G., Magaret, A.S., Prager, S.W., and Micks, E.A. (2020). The effect of testosterone on ovulatory function in transmasculine individuals. Am. J. Obstet. Gynecol. *223*, 229.e1–229.e8, ARTN 229.e1-e8. https://doi.org/10.1016/j.ajog.2020.01.059.
6. Stark, B., Hughto, J.M.W., Charlton, B.M., Deutsch, M.B., Potter, J., and Reisner, S.L. (2019). The contraceptive and reproductive history and planning goals of trans-masculine adults: a mixed-methods study. Contraception *100*, 468–473. https://doi.org/10.1016/j.contraception.2019.07.146.
7. Light, A.D., Obedin-Maliver, J., Sevelius, J.M., and Kerns, J.L. (2014). Transgender men who experienced pregnancy after female-to-male gender transitioning. Obstet. Gynecol. *124*, 1120–1127. https://doi.org/10.1097/AOG.0000000000000540.
8. Ikeda, K., Baba, T., Noguchi, H., Nagasawa, K., Endo, T., Kiya, T., and Saito, T. (2013). Excessive androgen exposure in female-to-male transsexual persons of reproductive age induces hyperplasia of the ovarian cortex and stroma but not polycystic ovary morphology. Hum. Reprod. *28*, 453–461. https://doi.org/10.1093/humrep/des385.
9. Khalifa, M.A., Toyama, A., Klein, M.E., and Santiago, V. (2019). Histologic Features of Hysterectomy Specimens From Female-Male Transgender Individuals. Int. J. Gynecol. Pathol. *38*, 520–527. https://doi.org/10.1097/pgp.0000000000000548.
10. Lin, L.H., Hernandez, A., Marcus, A., Deng, F.-M., and Adler, E. (2022). Histologic Findings in Gynecologic Tissue From Transmasculine Individuals Undergoing Gender-Affirming Surgery. Arch. Pathol. Lab Med. *146*, 742–748. https://doi.org/10.5858/arpa.2021-0199-OA.
11. Loverro, G., Resta, L., Dellino, M., Edoardo, D.N., Cascarano, M.A., Loverro, M., and Mastrolia, S.A. (2016). Uterine and ovarian changes during testosterone administration in young female-to-male transsexuals. Taiwan. J. Obstet. Gynecol. *55*, 686–691. https://doi.org/10.1016/j.tjog.2016.03.004.
12. Miller, N., Bédard, Y.C., Cooter, N.B., and Shaul, D.L. (1986). Histological changes in the genital tract in transsexual women following androgen therapy. Histopathology *10*, 661–669.
13. SPINDER, T., SPIJKSTRA, J.J., VAN DEN TWEEL, J.G., BURGER, C.W., VAN KESSEL, H., Hompes, P.G., and Gooren, L.J. (1989). The Effects of Long Term Testosterone Administration on Pulsatile Luteinizing Hormone Secretion and on Ovarian Histology in Eugonadal Female to Male Transsexual Subjects. J. Clin. Endocrinol. Metab. *69*, 151–157. https://doi.org/10.1210/jcem-69-1-151.
14. Coşkun, A.D.E., Koç, N., Vural, F., and Dönmez, E.E. (2023). Ovarian Histology of Removed Ovaries After Gender-Affirming Testosterone Therapy. Int. J. Surg. Pathol. *31*, 1093–1098. https://doi.org/10.1177/10668969231185087.
15. Borrás, A., Manau, M.D., Fabregues, F., Casals, G., Saco, A., Halperin, I., Mora, M., Goday, A., Barral, Y., and Carmona, F. (2021). Endocrinological and ovarian histological investigations in assigned female at birth transgender people undergoing testosterone therapy. Reprod. Biomed. Online *43*, 289–297. https://doi.org/10.1016/j.rbmo.2021.05.010.
16. De Roo, C., Lierman, S., Tilleman, K., Peynshaert, K., Braeckmans, K., Caanen, M., Lambalk, C.B., Weyers, S., T'Sjoen, G., Cornelissen, R., and De Sutter, P. (2017). Ovarian tissue cryopreservation in female-to-male transgender people: insights into ovarian histology and physiology after prolonged androgen treatment. Reprod. Biomed. Online *34*, 557–566. https://doi.org/10.1016/j.rbmo.2017.03.008.
17. Futterweit, W., and Deligdisch, L. (1986). Histopathological effects of exogenously administered testosterone in 19 female to male transsexuals. J. Clin. Endocrinol. Metab. *62*, 16–21.
18. Grimstad, F.W., Fowler, K.G., New, E.P., Ferrando, C.A., Pollard, R.R., Chapman, G., Gray, M., and Gomez Lobo, V. (2020). Ovarian Histopathology in Transmasculine Persons on Testosterone: A Multicenter Case Series. J. Sex. Med. *17*, 1807–1818. https://doi.org/10.1016/j.jsxm.2020.05.029.
19. Grynberg, M., Fanchin, R., Dubost, G., Colau, J.C., Brémont-Weil, C., Frydman, R., and Ayoubi, J.M. (2010). Histology of genital tract and breast tissue after long-term testosterone administration in a female-to-male transsexual population. Reprod. Biomed. Online *20*, 553–558. https://doi.org/10.1016/j.rbmo.2009.12.021.
20. PACHE, T.D., CHADHA, S., Gooren, L.J., Hop, W.C., JAARSMA, K.W., Dommerholt, H.B., and Fauser, B.C. (1991). Ovarian morphology in long-term androgen-treated female to male transsexuals. A human model for the study of polycystic ovarian syndrome? Histopathology *19*, 445–452. https://doi.org/10.1111/j.1365-2559.1991.tb00235.x.
21. Moseson, H., Fix, L., Ragosta, S., Forsberg, H., Hastings, J., Stoeffler, A., Lunn, M.R., Flentje, A., Capriotti, M.R., Lubensky, M.E., and Obedin-Maliver, J. (2021). Abortion experiences and preferences of transgender, nonbinary, and gender-expansive people in the United States. Am. J. Obstet. Gynecol. *224*, 376.e1–376.e11. https://doi.org/10.1016/j.ajog.2020.09.035.
22. Hembree, W.C., Cohen-Kettenis, P.T., Gooren, L., Hannema, S.E., Meyer, W.J., Murad, M.H., Rosenthal, S.M., Safer, J.D., Tangpricha, V., and T'Sjoen, G.G. (2017). Endocrine Treatment of Gender-Dysphoric/Gender-Incongruent Persons: An Endocrine Society Clinical Practice Guideline. J. Clin. Endocrinol. Metab. *102*, 3869–3903. https://doi.org/10.1210/jc.2017-01658.
23. Heeren, A.M., van Iperen, L., Klootwijk, D.B., de Melo Bernardo, A., Roost, M.S., Gomes Fernandes, M.M., Louwe, L.A., Hilders, C.G., Helmerhorst, F.M., van der Westerlaken, L.A.J., and Chuva de Sousa Lopes, S.M. (2015). Development of the follicular basement membrane during human gametogenesis and early folliculogenesis. BMC Dev. Biol. *15*, 4. https://doi.org/10.1186/s12861-015-0054-0.
24. Murphy, B.D. (2000). Models of Luteinization. Biol. Reprod. *63*, 2–11. https://doi.org/10.1095/biolreprod63.1.2.
25. Kirkendoll, S.D., and Bacha, D. (2023). Histology, Corpus Albicans. In StatPearls (StatPearls Publishing Copyright © 2023, StatPearls Publishing LLC.).

STAR★METHODS

KEY RESOURCES TABLE

REAGENT or RESOURCE	SOURCE	IDENTIFIER
Biological samples		
Transgender and gender diverse people's ovarian tissue samples after gender affirming oophorectomy	Amsterdam UMC, the Netherlands	NA
Software and algorithms		
SPSS version 20.0	IBM Corporation	NA
CaseViewer	3D HISTECH Ltd.	NA

RESOURCE AVAILABILITY

Lead contact

Further information and requests for resources should be directed to and will be fulfilled by the lead contact, Norah van Mello (n.vanmello@amsterdamumc.nl)

Materials availability

This study did not generate new unique reagents.

Data and code availability

- Data reported in this paper will be shared by the lead contact upon request.
- This paper does not report original code.
- Any additional information required to reanalyze the data reported in this paper is available from the lead contact upon request.

EXPERIMENTAL MODEL AND STUDY PARTICIPANT DETAILS

Study design and population

This prospective cohort study was executed in the Center of Expertise on Gender at the Amsterdam UMC and ovarian tissue analysis was performed at Leiden University Medical Center (LUMC).

All trans masculine people scheduled for oophorectomy at the Center of Expertise on Gender were approached for participation in this study between May 2019 and May 2022. Demographic characteristics, as well as medical histories, were collected from the medical records.

Trans masculine people at the Center of Expertise on Gender are eligible for oophorectomy after adequate gender affirming hormone therapy with testosterone for at least one year. In line with the European protocol, testosterone is administered trans dermally or intramuscularly and is considered adequate when within a cis gender male serum range (ref. 9,0–30,0 nmol/L).[22] In our center, this is achieved by means of testosterone administration dermally [Androgel, Besins International] (25 or 50 mg, daily) or via intramuscular injection [Sustanon, Aspen Pharma Trading Limited] (250 mg per 2–4 weeks) or [Nebido, Bayer] (1 g, per 10–14 weeks). In line with international guidelines,[1] our center performs three monthly lab tests when starting (a new type of) testosterone for a year, followed by (bi-)annual lab tests once the maintenance dose is attained.

Trans masculine people using other hormonal medication with an effect on ovulation, such as gonadotropin-releasing hormone agonist (GnRHa) or hormonal contraception at the time of oophorectomy, were excluded from this study cohort. People who underwent oocyte vitrification in the year prior to GAS were also excluded from this study cohort.

Ethical approval

This study did not fall within the scope of the Dutch Medical Research with Human Subjects Law (WMO) and was proved exempt by a letter of no objection by the local Medical Ethical Committee of the LUMC (B18.029) and locally approved by the board of directors at the Amsterdam UMC, location VUmc. Written informed consent was obtained from all participants.

METHOD DETAILS

Laboratory procedures

After oophorectomy, the ovaries were kept in NaCl 0.9% on ice and transported for histological analyses. Upon arrival, the ovaries were bisected, imaged and fixed in 4% paraformaldehyde (Merck, Germany) diluted in phosphate buffered saline (PBS) overnight at

4°C. Thereafter, the tissue was washed overnight in PBS, transferred to 70% Ethanol and embedded in paraffin using a Shandon Excelsior tissue processor (Thermo Scientific, Altrincham, UK). After embedding, the tissue was sectioned (5 μm) using an RM2065 microtome (Leica Instruments GmbH, Wetzlar, Germany) and paraffin sections were stained with hematoxylin and eosin (HE) as described elsewhere.[23]

QUANTIFICATION AND STATISTICAL ANALYSIS

Imaging and quantification criteria

Histological images were used to quantify ovulatory function, including the presence of small antral follicles, ovulatory follicles, corpus luteum and corpus albicans. The HE-stained slides were scanned with a Panoramic 250 digital scanner (3DHISTECH Ltd., Budapest, Hungary) and analyzed using CaseViewer software (3DHISTECH Ltd., Budapest, Hungary).

Antral follicles are characterized by their cystic morphology, a fluid-filled cavity in between the granulosa cells in the ovarian tissue. We defined antral follicles as small (1–5 mm in diameter) or as ovulatory (>5 mm, containing a ruptured area that could be observed on the surface of the ovary as well as from the histological sections). A corpus luteum, a temporary endocrine structure that forms in the ovary after ovulation, was defined by the presence of a large yellow structure (≥10 mm) showing luteinized granulosa cells on the histological sections.[24] A corpus albicans, a fibrous structure that forms in the ovary after the regression of the corpus luteum, was defined as a non-vascularized, white structure formed by dense connective tissue and low cellular density.[25] Even though corpus luteum and corpus albicans can remain in the ovary for several months, all participants were >12 months on adequate testosterone treatment, therefore the presence of a corpus luteum and corpus albicans were also included as a sign of recent ovulation.

Statistical analysis

Statistical analyses were performed using SPSS version 20.0 (SPSS, Inc., Chicago, IL, USA). Baseline characteristics of the cohort are presented as mean with standard deviation (SD) when normally distributed, and as median with interquartile range (IQR) when non-normally distributed. Quantitative data are presented as number with percentage. Non-parametric Mann–Whitney U, Chi-square tests and Fishers exact tests were performed where appropriate. A p value ≤ 0.05 was considered statistically significant.

Cell Reports Medicine, Volume *5*

Supplemental information

One-third of amenorrheic transmasculine people on testosterone ovulate

Joyce D. Asseler, Julieta S. del Valle, Susana M. Chuva de Sousa Lopes, Marieke O. Verhoeven, Mariette Goddijn, Judith A.F. Huirne, and Norah M. van Mello

Table S1. Data on prior GnRHa users of study cohort, related to Table 1.

	Age at GAS, years	Age at start GnRHa, years	Total time on GnRHa, months	Indication GnRHa	Tanner stage at time start GnRHa	Time of GnRHa cessation prior to GAS, months	Histological signs of recent ovulatory activity
1	20	14	40	PS	Unknown	30	Yes, corpus luteum and corpus albicans
2	19	14	19	PS	M4P5	35	Yes, corpus luteum and corpus albicans
3	20	16	14	PS	M5P5	22	Yes, corpus albicans
4	21	17	9	PS	M5P5	40	Yes, corpus luteum
5	20	17	11	PS	M5P5	23	Yes, corpus luteum
6	18	13	47	PS	M3P3	9	No
7	18	16	19	PS	M5P5	8	No
8	21	17	26	PS	M5P5	18	No
9	19	17	12	PS	M5P5	25	No
10	28	26	4	B	N/a	18	No
11	19	11	49	PS	M4P5	42	No
12	21	17	12	PS	M5P5	40	No
13	19	16	36	PS	Unknown	7	No
14	19	12	50	PS	M3P2	31	No
15	18	12	55	PS	M3P4	19	No

Abbreviations used in table: GnRHa = gonadotropin-releasing hormone agonist, PS = puberty suppressant, N/a = not applicable, B: persistent blood loss.
